# Effects of resistance training on alleviating hypoxia‐induced muscle atrophy: Focus on acetylation of FoxO1

**DOI:** 10.1111/jcmm.18096

**Published:** 2023-12-27

**Authors:** Pengyu Fu, Rongxin Zhu, Weiyang Gao, Lijing Gong

**Affiliations:** ^1^ Key Laboratory of Physical Fitness and Exercise, Ministry of Education Beijing Sport University Beijing China; ^2^ Department of Physical Education Northwestern Polytechnical University Xi'an China; ^3^ Shanghai Research Institute of Sports Science Shanghai China; ^4^ School of Languages and Cultural Communication, English Department Xi’an Mingde Institute of Technology Xi’an China

**Keywords:** acetyl‐FoxO1, autophagy, hypoxia, muscle atrophy, resistance training

## Abstract

This study aims to explore the role of FoxO1 and its acetylation in the alleviation of hypoxia‐induced muscle atrophy by resistance training. Forty male Sprague–Dawley rats were randomly divided into four groups: normoxic control group (C), normoxic resistance training group (R), hypoxic control group (H) and hypoxic resistance training group (HR). Rats in R and HR groups were trained on an incremental weight‐bearing ladder every other day, while those in H and HR groups were kept in an environment containing 12.4% O_2_. After 4 weeks, muscles were collected for analysis. Differentiated L6 myoblasts were analysed in vitro after hypoxia exposure and plasmids transfection (alteration in FoxO1 acetylation). The lean body mass loss, wet weight and fibre cross‐sectional area of extensor digitorum longus of rats were decreased after 4 weeks hypoxia, and the adverse reactions above was reversed by resistance training. At the same time, the increase in hypoxia‐induced autophagy was suppressed, which was accompanied by a decrease in the expression of nuclear FoxO1 and cytoplasmic Ac‐FoxO1 by resistance training. The L6 myotube diameter increased and the expression of autophagic proteins were inhibited under hypoxia via intervening by FoxO1 deacetylation. Overall, resistance training alleviates hypoxia‐induced muscle atrophy by inhibiting nuclear FoxO1 and cytoplasmic Ac‐FoxO1‐mediated autophagy.

## 1 INTRODUCTION

Oxygen availability is reduced during ascent to high altitudes or in environments with low atmospheric oxygen percentage.^1^ The production of red blood cells increases after hypoxia exposure for a period of time to promote aerobic metabolism in the plain area, which is a good training method for endurance athletes and is called high‐altitude training in exercise physiology.^2^ However, in response to hypoxia, skeletal muscle proteolysis is initiated, which leads to muscle wasting.^3^, ^4^ A series of studies reported up to 15%–20% reductions in fibre cross‐sectional area (FCSA)^5^, ^6^ and proved that this phenomenon is independent of the reduction of food intake in a hypoxic environment.^7^ Although this protective mechanism can reduce the oxygen diffusion distance, the decrease in muscle strength will reduce the training effect of athletes^8^ and affect the health of tourists in high‐altitude areas.^9^ High‐altitude expedition climbers who engage in prolonged periods of intense physical activity, often over weeks or months, experience considerably less muscle atrophy.^10^ The ratio of capillaries/muscle fibres increased in cross‐country skiers who trained at an altitude of 2700 m for 2 weeks.^11^ Furthermore, exercise training in a hypoxic environment has emerged as a new and promising therapeutic avenue to improve certain metabolic and cardiovascular diseases.^12^ Thus, exploring the effect of hypoxic exercise on skeletal muscle protein metabolism will be an interesting and important research direction.

Our previous study found that, 10 days resistance training at an altitude of 3700 m, muscle atrophy was effectively inhibited in male college students; changes in forkhead box protein O1 (FoxO1) participated in the process, based on muscle biopsy and gene expression microarray analyses. FoxO1, is a nuclear transcription factor, involved in the regulation of skeletal muscle mass,^13^ and its expression increases in muscle atrophy induced by fasting, cachexia, aging, muscle disuse, diabetes, etc.^14^ The body weight and muscle mass of mice were reduced by specific overexpression of FoxO1 in muscles.^15^ FoxO1 could promote muscle proteolysis by regulating the autophagy lysosome pathway (ALP) and the ubiquitin proteasome pathway (UPS) in normoxia.^16^, ^17^ An increase in protein breakdown is an important factor for hypoxia‐induced muscle atrophy,^18^, ^19^ and the role of FoxO1 in this process needs further study. The relationship between FoxO1 expression and muscle fibre types, and its selectivity to hypoxia and/or resistance training should be further explored.

ALP is an important protein breakdown pathway that occurs in the cytoplasm, and it also regulates the expression of muscle‐specific F‐box protein (MAFbx/Atrogin‐1) and muscle‐specific ring finger 1 (MuRF1) in the UPS pathway.^20^ Excessive autophagy is an essential regulator of muscle atrophy caused by diabetes, acquired immunodeficiency syndrome (AIDS) and chronic obstructive pulmonary disease (COPD).^21^ The effect of exercise is considered to be involved in the autophagy pathway on muscles.^22^ Cytoplasmic FoxO1 is an important mediator of autophagy, which contradicts previous report that FoxO1 is inactivated in the nucleus after phosphorylation modification. Tumour cells undergo autophagy during oxidative stress or serum starvation, and this process is regulated by acetylation of FoxO1 (Ac‐FoxO1). FoxO1 dissociates from deacetylase sirtuin 2 (SIRT2) under stress stimulation, and is acetylated in the cytoplasm, then Ac‐FoxO1 binds to the key autophagy protein autophagy associated gene 7 (Atg7) to activate autophagy.^23^, ^24^ The role of resveratrol in improving chronic kidney disease‐induced muscle atrophy is related to the involvement of SIRTs in reducing the acetylation of FoxO1.^25^ This finding provides a premise to explore the role of FoxO1 acetylation‐mediated autophagy pathway in regulating muscle atrophy. Increasing number of studies showed that the regulatory scope of protein acetylation by exercise is widespread in skeletal muscles.^26^, ^27^ Thus, we aimed to confirm the role of FoxO1 acetylation/deacetylation modification in the alleviation of hypoxia‐induced muscle atrophy by resistance training through in vitro and in vivo experiments.

## 2 MATERIALS AND METHODS

### 2.1 Animals

All experimental protocols were approved by the Institutional Animal Care and Use Committee of Beijing Sport University (Ref. No: 2017009). Forty male Sprague–Dawley rats (8‐week‐old, weighing 236.4 ± 10.69 g) were purchased from Charles River Development, Inc. (Beijing, China), and randomly divided into four groups: normoxic control (Group C), normoxic resistance ladder training (Group R), hypoxic control (Group H) and hypoxic resistance ladder training (Group HR), with 10 rats per group. All rats were housed indoors under a temperature of 22 ± 2°C, humidity of 40%–70%, 12‐h light/dark cycle and given ad libitum access to deionized water and food.

### 2.2 Training protocol

Rats in Groups R and HR received resistance training, which included ladder climbing with increasing load. The ladder was 1.2 m long, and oriented at 85° relative to the ground. Rats were placed at the bottom of the ladder and accepted appropriate stimulation at the tail to impel them from climbing to the top (completed within 10 s) for one set. A weight‐bearing method was conducted as following: applying adhesive tape to the tail‐head of rats, tying a rubber band around the tape, and adjusting the weight‐bearing device (i.e. increasing or decreasing the number of steel balls in 50 mL centrifuge tubes). Pre‐adaption training was performed daily with 0%–50% increments of the rat body weight at the first week, and 50%–130% weight‐bearing training was carried out every other day in the next formal training (training intensity was five times × three sets, rest for 1 min between sets) for 4 weeks^28^ (the weight‐bearing protocol is shown in Table 1). Rats in Groups H and HR were kept in a room with an oxygen concentration of 12.4% (simulating an approximate altitude of 4000 m) for 4 weeks.

**TABLE 1:** Weight‐bearing protocol (*n* = 40).

Time/day	Pre‐training	Formal training								
	1–7	1	3	5	7	9	11	13	15	17–27
Weight‐bearing (body weight%)	0–50	50	60	70	80	90	100	110	120	130
Weight of R/gram	0–121	121	153	188	217	257	294	323	376	422–485
Weight of HR/gram	0–115	115	145	177	196	234	272	310	353	395–442

Abbreviations: HR, hypoxic resistance ladder training; R, normoxic resistance training group.

### 2.3 Food intake, body weight and body composition

The food intake and body weight of rats were recorded every day. At 12 h after the last intervention, the rats were anaesthetized by intraperitoneal injection of 3% sodium pentobarbital (3 mL/kg). Body composition was detected by dual‐energy x‐ray absorptiometry (DEXA, XR‐46, Norland, USA).

### 2.4 Tissue collection and preservation

At 24 h after the last training session, the rats were anaesthetized and sacrificed by blood sampling from their abdominal aorta after 12 h fasting. The musculi soleus (SOL, slow‐twitch muscle), gastrocnemius (GAS, mixed‐twitch muscle) and extensor digitorum longus (EDL, fast‐twitch muscle) were isolated and weighed. Muscles were kept in 4% paraformaldehyde fixative, or quick‐frozen in liquid nitrogen and stored at −80°C.

### 2.5 Cell culture and intervention

L6 myoblasts were cultured in Dulbecco's Modified Eagle's Medium containing 10% fatal bovine serum, and then replaced with medium containing 2% horse serum to differentiate when the cells reached 80%–90% confluence on the flask bottom. The culture environment was 21% O_2_, 5% CO_2_ and 37°C, and the medium was changed every other day. On the sixth–seventh day of differentiation, the myoblasts became elongated and spindle‐shaped, indicating myotube formation. Differentiated L6 myotubes were divided into the Normoxia and Hypoxia Groups. The Hypoxia Group was exposed to 1% O_2_ for 6 h in a hypoxia chamber.

### 2.6 Plasmid design and virus transfection

FoxO1 overexpression and lysine (Lys) mutant plasmids were constructed (simulated to maintain acetylation and deacetylation). Lys at 239, 242, 256, 259 and 268 sites {according to the UniProt database (KB‐G3V7R4 [FoxO1 Rat]), all sites could promote the localisation of FoxO1 in the cytoplasm and change its acetylation state} mutated into glutamine (Glu) (K to Q) to simulate acetylation and into arginine (Arg) (K to R) to simulate deacetylation.^29^, ^30^, ^31^ The following plasmids were constructed and packed with adenovirus vector: (1) enhanced green fluorescent protein (EGFP); (2) rFoxO1: pAV[Exp]‐CMV > rFoxO1[NM_001191846.2](ns):T2A:EGFP; (3) K to Q: pAV[Exp]‐CMV > {rFoxO1[NM_001191846.2] × 5 K > Q(ns)}:T2A:EGFP; (4) K to R: pAV[Exp]‐CMV > {rFoxO1[NM_001191846.2] × 5 K > R(ns)}:T2A:EGFP, and then transfected into L6 myotubes for 6 h. Cells were collected after 48 h.

### 2.7 Immunofluorescence (IF) microscopy

Paraffin sections were washed with a gradient series, rinsed with distilled water, and subjected to antigen retrieval, quenching autofluorescence, and bovine serum albumin blocking. The sections were then incubated with Laminin (Cat. No. ab207612, Abcam, UK), FoxO1 (Cat. No. ab52857, Abcam, UK), Ac‐FoxO1 (Cat. No. Orb543702, Biorbyt, UK) and Myosin antibodies (Cat. No. ab124205, Abcam, UK) overnight at 4°C, followed by secondary antibodies (Cat. No. GB21303/926–32,210, Servicebio, China) after washing with buffer at room temperature for 50 min. Cell nuclei were counterstained with DAPI, mounted onto slides, and photographed under a microscope. Five different fields of view were selected from the centre and four corners of each slice, and Image pro Plus 6.0 (Media Cybernetics, Inc. USA) software was used for blind analysis by a third person. The FCSA of muscle were calculated, and the integrated optical density (IOD) value of the selected area was measured to indicate immunopositivity. The percentages of FoxO1 and Ac‐FoxO1 nuclear localisation were calculated.

### 2.8 Western blot

Total protein was extracted and quantified with a bicinchoninic acid assay kit (Thermo Fisher Scientific, USA). Cytoplasmic and nuclear proteins were extracted and quantified using Minute™ Cytoplasmic and Nuclear Fractionation kit (Cat. No. SC‐003 Invent Biotechnologies, USA). Proteins samples were loaded on 4–12% Bis‐Tris and 3–8% Tris Acetate gradient gels (NW04125/EA03755, Invitrogen, USA). The proteins were then transferred onto nitrocellulose (NC) membrane. The target proteins were blocked with Odyssey Blocking buffer (LI‐COR, USA) and probed overnight at 4°C by using the antibodies of SIRT2 (Cat. No. ab211033, Abcam, UK), Ras‐related GTP‐binding protein 7 (Rab7) (Cat. No. ab137029, Abcam, UK), Atg7 (Cat. No. ab133528, Abcam, UK), microtubule‐associated protein light chain 3 (LC3) (Cat. No. NB100‐2220, Novusbio, USA), p62/SQSTM1 (Cat. No. ab109012, Abcam, UK), Atrogin‐1 (Cat. No. ab74023, Abcam, UK), MuRF1 (Cat. No. ab172479, Abcam, UK), Ubiquitin (Cat. No. sc‐8017, Santa cruz, USA), TATA binding protein (TBP) (Cat. No. 66166‐1‐Ig, Proteintech, China) and α‐tubulin (Cat. No. T6074, Sigma, Germany). On the following day, the membranes were washed with Tris‐buffered saline (TBS) containing 1% Tween‐20 and incubated with goat anti‐rabbit/mouse IgG (Cat. No. 926–68,071/926–32,210, LI‐COR, USA) at 25°C for 1 h. The membranes were washed twice with TBS, and signals were detected using the near‐infrared spectroscopy detection system (Odyssey CLX, LI‐COR). All bands were analysed semi‐quantitatively using Image Studio ver 5.2.

### 2.9 Statistical analyses

Statistical analysis was performed using SPSS 22.0. All data are presented as mean ± standard error of the mean. Three and two‐way ANOVA analysis was used in in vivo; independent sample *t*‐test and one‐way ANOVA analysis were used in in vitro. The significance level was set at *p* < 0.05.

## 3 RESULTS

### 3.1 Muscle atrophy degree of rats

**FIGURE 1:**
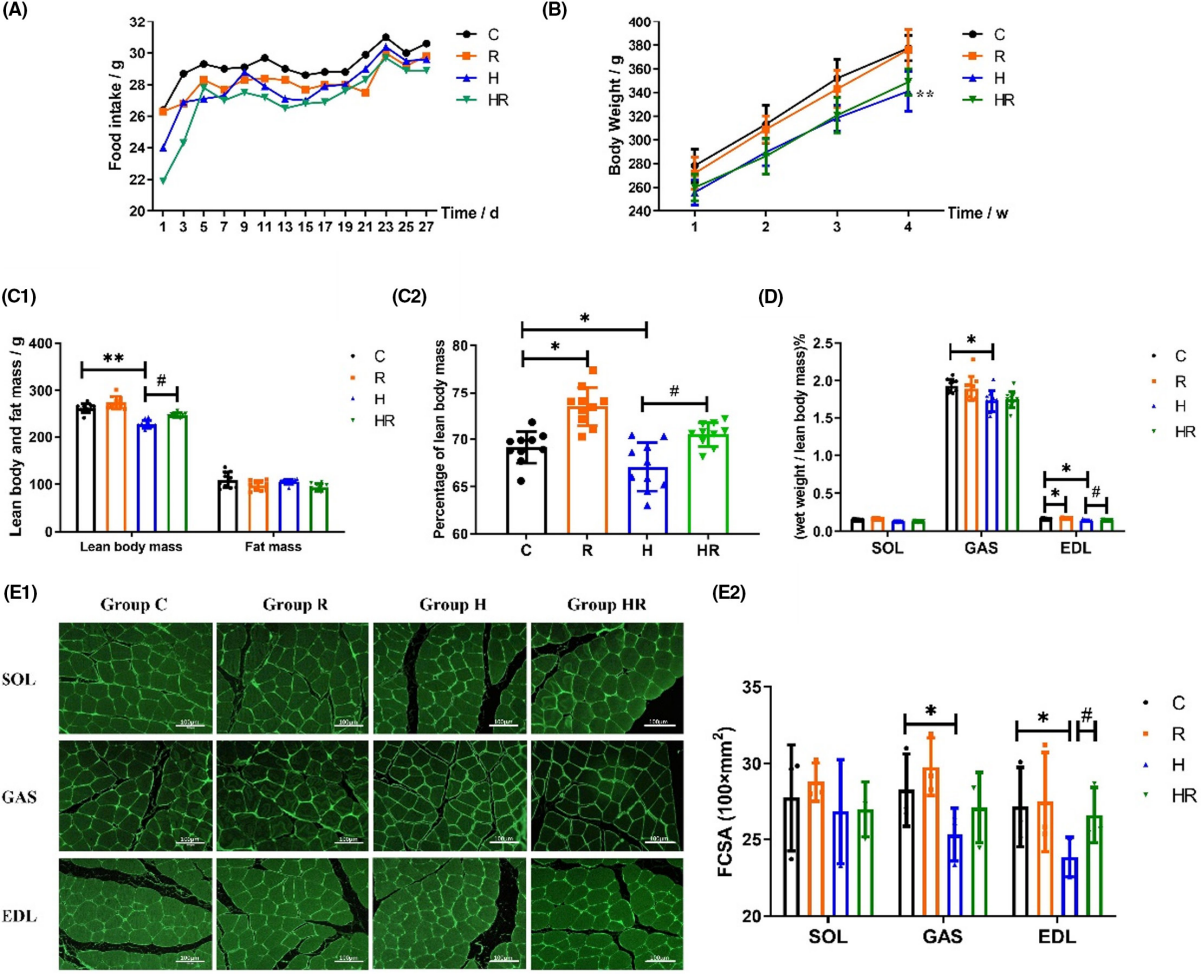
The degree of muscle atrophy of rats (three‐way ANOVA for A and B; two‐way ANOVA for C–E). The food intake every other day during the intervention (*n* = 10) (A). The weekly body weight during the intervention (*n* = 10) (B). The lean body mass and the fat mass (C1), the percentage of lean body mass (C2), the percentage of SOL, GAS and EDL wet weight (wet weight / lean body mass × 100%) (D) after the intervention (*n* = 10). Laminin IF staining (E1) and the FCSA (E2) of SOL, GAS and EDL after the intervention (*n* = 3). *Significant different from Group C; ^#^Significant different from Group H (*p* < 0.05). d, day; w, week; g, gram; SOL, soleus muscle; GAS, gastrocnemius muscle; EDL, extensor digitorum longus muscle; C, normoxic control; R, normoxic resistance ladder training; H, hypoxic control; HR, hypoxic resistance ladder training.

During the intervention, the food intake of the hypoxic groups (Groups H and HR) was lower than that of the normoxic groups (Groups C and R) in the early stage of intervention, and it tended to be close in the late stage (Figure 1A). At the end of the intervention, the body weight of rats in Group H was lower than that in Group C (*p* < 0.01) (Figure 1B). After the intervention, the lean body mass of rats was significantly lower in Group H than in Group C (*p* < 0.01) and higher in Group HR than in Group H (*p* < 0.05); however, no difference in fat mass index was observed (Figure 1C1). The percentage of lean body mass (lean body mass/body weight) in Group R was significantly higher than that in Group C; moreover, it was lower than Group H than in Group C and higher in Group HR than in Group H (*p* < 0.05) (Figure 1C2). The percentage of GAS and EDL wet weight (wet weight/lean body mass) in Group H was significantly lower than that in Group C. The percentage of EDL wet weight in Group R was significantly higher than that in Group C, and the value in Group HR was significantly higher than that in Group H (*p* < 0.05). No difference in SOL wet weight was detected between groups (Figure 1D). Laminin IF staining was used to calculate FCSA (Figure 1E1). The FCSA of GAS and EDL in Group H was significantly lower than that in Group C, and the FCSA of EDL in Group HR was significantly higher than that in Group H (*p* < 0.05) (Figure 1E2). The results suggest that resistance training has a significant effect in relieving hypoxia‐induced muscular atrophy in EDL. Thus, EDL was selected for subsequent studies.

### 3.2 FoxO1 and its acetylation levels in EDL of rats

**FIGURE 2:**
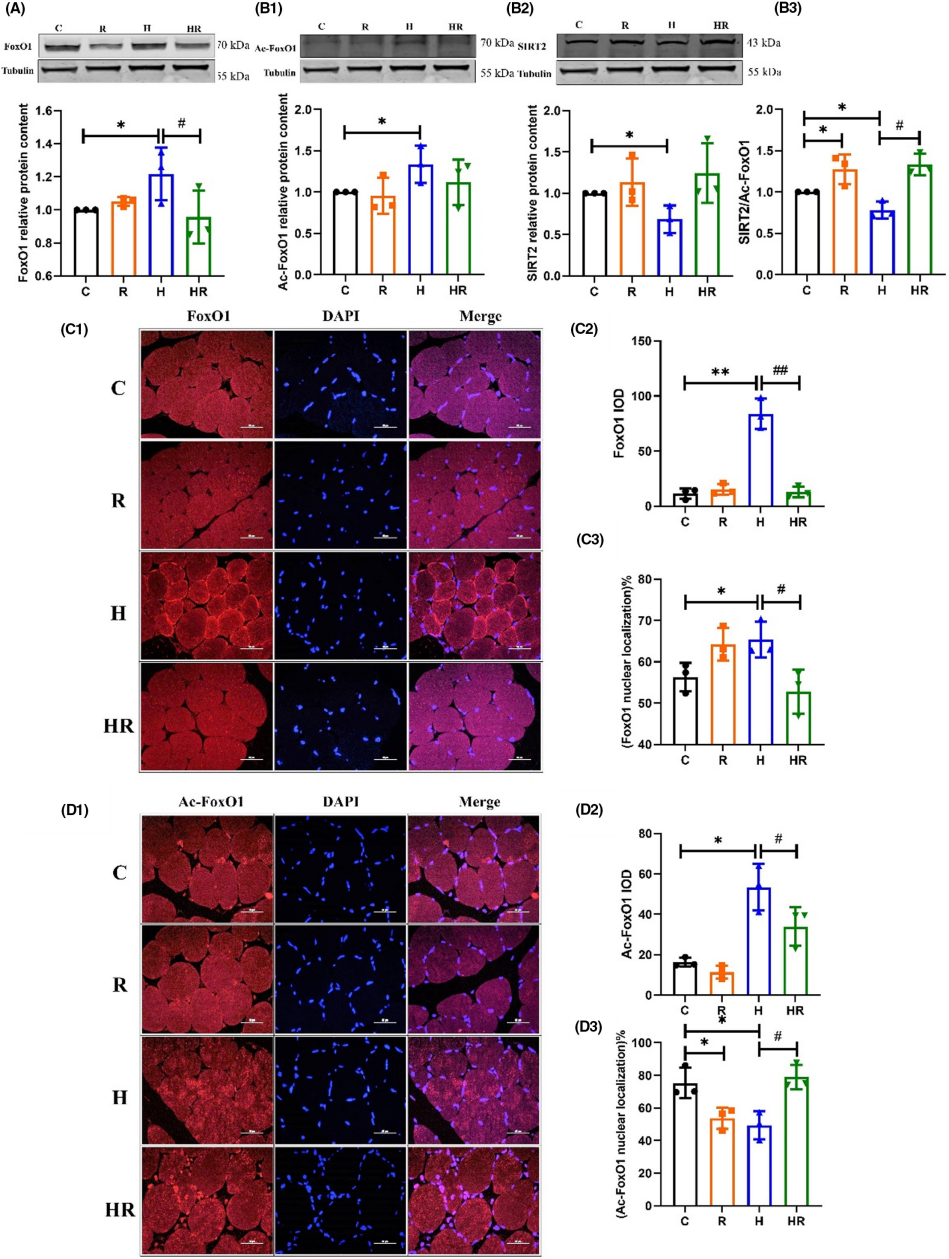
The levels of FoxO1 and its acetylation in EDL of rats after the intervention (*n* = 3) (two‐way ANOVA). The proteins expression of FoxO1 (A), Ac‐FoxO1 (B1), SIRT2 (B2), and the ratio of SIRT2/Ac‐FoxO1 (B3). FoxO1 (C1) and Ac‐FoxO1 (D1) IF staining. FoxO1 and Ac‐FoxO1 are red, DAPI‐stained nuclei are blue. The integrated optical density (IOD) of FoxO1 (C2) and Ac‐FoxO1 (D2). The percentage of FoxO1 (C3) and Ac‐FoxO1 (D3) nuclear localisation. *Significant different from Group C; ^#^Significant different from Group H (*p* < 0.05).

After the intervention, the expression levels of FoxO1, Ac‐FoxO1 and SIRT2 of EDL in Group H were significantly higher than those in Group C, while the expression of FoxO1 in Group HR was significantly lower than that in Group H (*p* < 0.05) (Figure 2A,B1,B2). The ratio of deacetylase and Ac‐FoxO1 reflects the deacetylase activity, which becomes stronger with increasing ratio. The ratio of SIRT2/Ac‐FoxO1 was higher in Group R than in Group C, lower in Group H than that in Group C, and higher in Group HR than in Group H, lower in Group H (*p* < 0.05) (Figure 2B3). The IOD values of FoxO1 (*p* < 0.01) and Ac‐FoxO1 (*p* < 0.05) in Group H were higher than those in Group C, and the values in Group HR were lower than those in Group H by IF staining IOD (*p* < 0.01 and *p* < 0.05, respectively) (Figure 2C1,C2,D1,D2). The percentage of FoxO1 nuclear localisation was higher in Group H than in Group C, and lower in Group HR than in Group H (*p* < 0.05) (Figure 2C1,C3). The percentage of Ac‐FoxO1 nuclear localisation was lower in Groups R and H than in Group C, but higher in Group HR than in Group H (*p* < 0.05) (Figure 2D1,D3).

**FIGURE 3:**
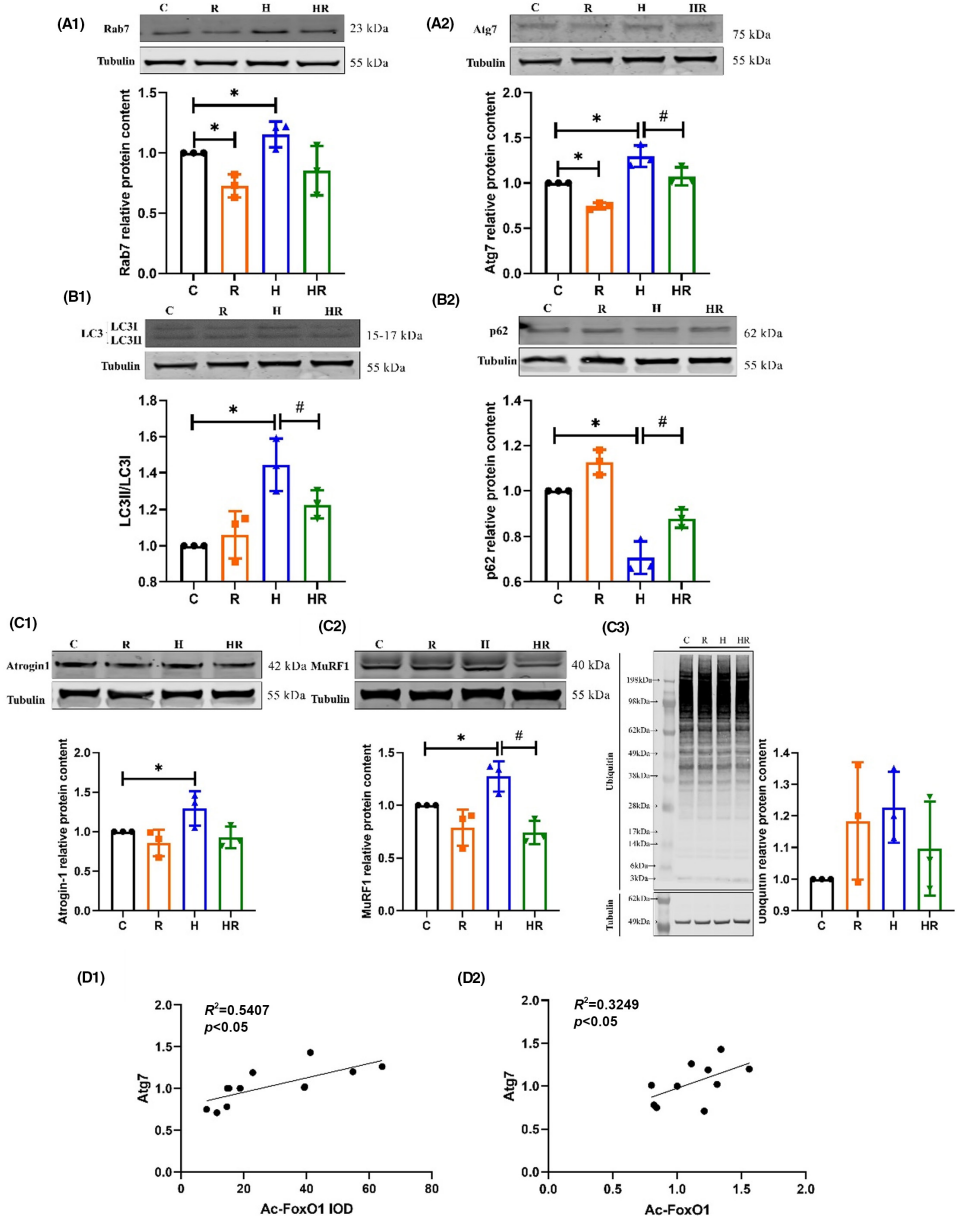
The levels of ALP and UPS in EDL of rats after the intervention (*n* = 3) (two‐way ANOVA). The proteins expression of Rab7 (A1), Atg7 (A2), p62 (B2), Atrogin‐1 (C1), MuRF1 (C2) and Ub (C3) and the ratio of LC3II/I (B1). the IOD of Ac‐FoxO1 (D1) and the protein expression (D2) of Atg7. *Significant different from Group C; ^#^ Significant different from Group H (*p* < 0.05).

### 3.3 ALP and UPS levels in EDL of rats

The protein contents of Rab7, Atg7, LC3II/I and p62 were used to indicate ALP levels. After the intervention, the expression levels of Rab7 and Atg7 in Group R was lower than those in Group C, and the values in Group H were higher than those in Group C; the expression of Atg7 in Group HR was higher than that in Group H (*p* < 0.05) (Figure 3A1,A2). The ratio of LC3II/I was higher in Group H than in Group C, and lower in Group HR than in Group H (*p* < 0.05) (Figure 3B1). The expression of p62 in Group H was lower than that in Group C, but the level in Group HR was higher than that in Group H (*p* < 0.05) (Figure 3B2). The contents of Atrogin‐1 and MuRF1 were used to indicate UPS levels. The expression levels of Atrogin‐1 and MuRF1 were higher in Group H than those in Group C, and that of MuRF1 in Group HR was lower than that in Group H (*p* < 0.05) (Figure 3C1,C2). The expression of Ubiquitin was assayed to determine the overall protein ubiquitination levels. No significant difference was found in the ubiquitin levels among groups (Figure 3C3). ALP might play a critical role during this process. A significant correlation was found among Atg7 protein content, Ac‐FoxO1 protein content (Figure 3D1) and Ac‐FoxO1 IOD (Figure 3D2).

### 3.4 FoxO1 and its acetylation levels in cytoplasm and nucleus of L6 myotubes

**FIGURE 4:**
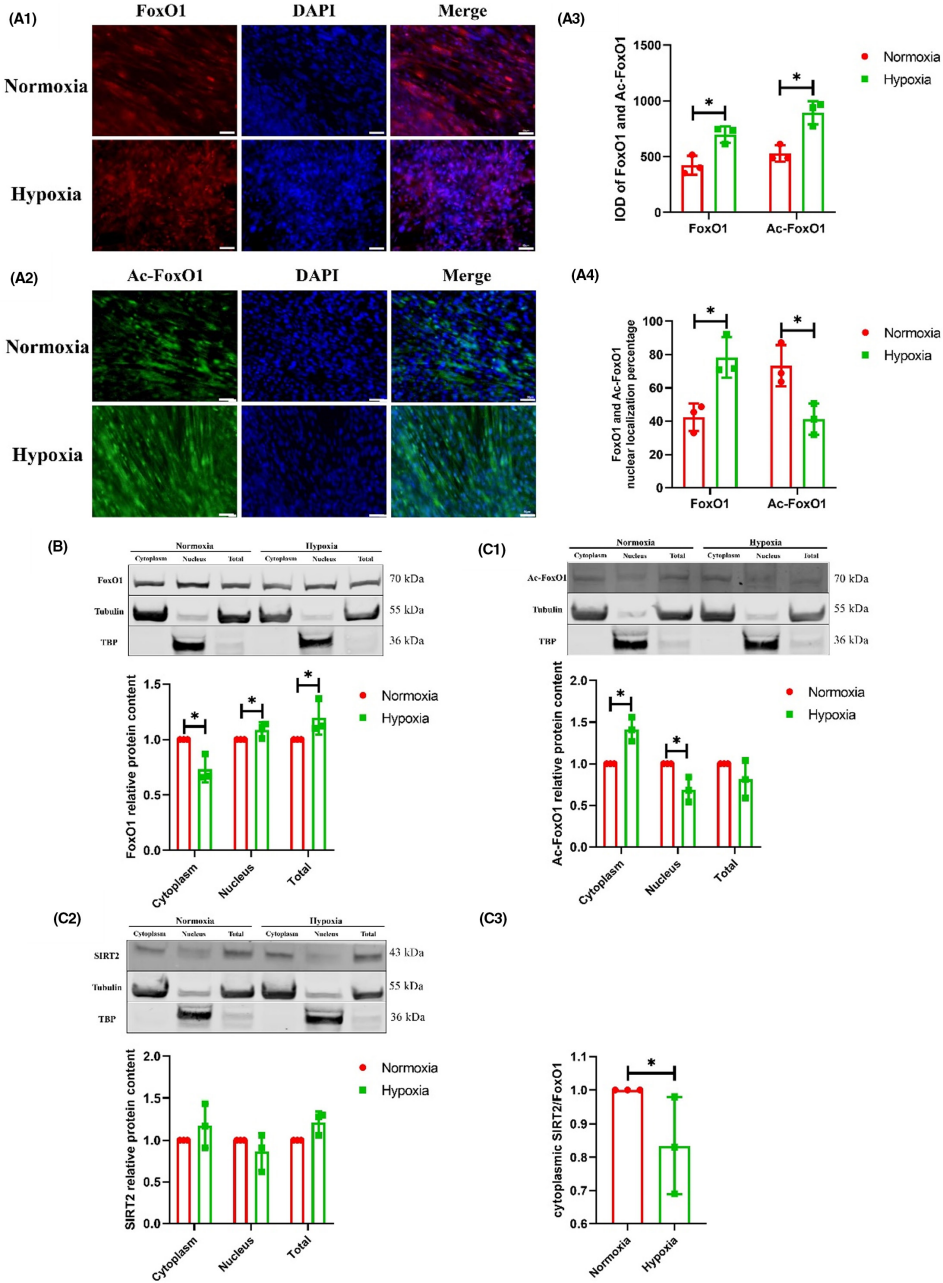
The levels of FoxO1 and its acetylation in cytoplasm and nucleus of L6 myotubes (*n* = 3) (independent sample *t*‐test). FoxO1 (A1) and Ac‐FoxO1 (A2) IF staining. FoxO1 is red, Ac‐FoxO1 is green, DAPI‐stained nuclei are blue. The IOD of FoxO1 and Ac‐FoxO1 (A3). The percentage of FoxO1 and Ac‐FoxO1 nuclear localisation (A4). The proteins expression of cytoplasmic, nuclear and total FoxO1 (B), Ac‐FoxO1 (C1) and SIRT2 (C2). The ratio of SIRT2/Ac‐FoxO1 in cytoplasm (C3). *Significant different from normoxia (*p* < 0.05).

After the intervention, the IOD values of FoxO1 and Ac‐FoxO1 under hypoxia exposure were higher than those under normoxia (*p* < 0.05) (Figure 4A1,A2,A3). In nuclear localisation, the percentage of FoxO1 under hypoxia exposure was higher than that under normoxia; the percentage of Ac‐FoxO1 under hypoxia exposure was lower than that under normoxia (*p* < 0.05) (Figure 4A1,A2,A4). The expression of cytoplasmic FoxO1 under hypoxia exposure was lower than that under normoxia, the expression of nuclear and total FoxO1 (*p* < 0.05) (Figure 4B). The expression of cytoplasmic Ac‐FoxO1 under hypoxia exposure was higher than that under normoxia, and the expression of nuclear FoxO1 under hypoxia exposure was lower than that under normoxia (*p* < 0.05) (Figure 4C1). No difference in SIRT2 level was found in the cytoplasm between hypoxia and normoxia exposure, as well as in nucleus and total cells (Figure 4C1). The ratio of SIRT2/Ac‐FoxO1 in cytoplasm under hypoxia exposure was lower than that under normoxia (*p* < 0.05) (Figure 4C3).

### 3.5 Effect of FoxO1 acetylation on L6 myotube diameter and autophagy level

**FIGURE 5:**
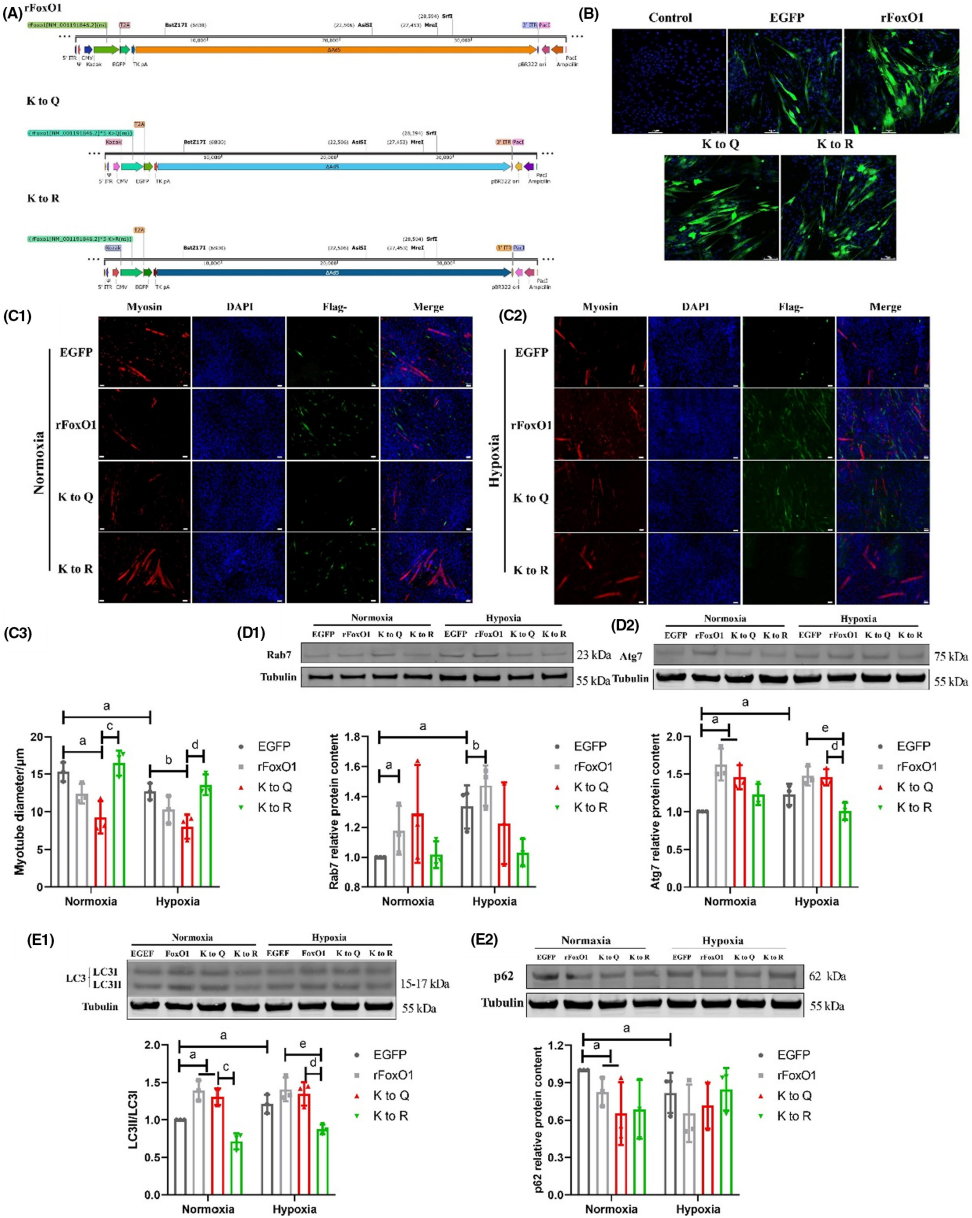
The effect of FoxO1 acetylation on L6 myotube diameter and autophagy level (*n* = 3) (Independent sample t‐test was used for comparison between normoxia and hypoxia group, and one‐way ANOVA was used for comparison between different plasmid groups in normal/hypoxia group). FoxO1 overexpression and Lysine mutant plasmid (simulated to maintain acetylation and deacetylation state) (A). Plasmids have been successfully transfected into the myotube (B). Myosin IF staining in normoxia (C1) and hypoxia (C2). Myosin is red, DAPI‐stained nuclei is blue, fluorescent marker is green. The myotube diameter (C3). The proteins expression of Rab7 (D1), Atg7 (D2), p62 (E2) and the ratio of LC3II/I (E1). ^a^Significant different from Group EGFP in normoxia; ^b^Significant different from Group EGFP in hypoxia; ^c^Significant different from Group K to Q in normoxia; ^d^Significant different from Group K to Q in hypoxia; ^e^Significant different from Group rFoxO1 in hypoxia (*p* < 0.05).

FoxO1 overexpression and lysine mutant plasmids (simulated to maintain acetylation and deacetylation state) are evaluated and shown in Figure 5A. After 48 h, EGFP, rFoxO1, K to Q, K to R plasmids were successfully transfected into the myotube (Figure 5B). For the myotube diameter, Group EGFP under hypoxia was lower than that under normoxia; Group K to Q was lower than Group EGFP, Group K to R was lower than Group K to Q in both normoxia and hypoxia exposure (*p* < 0.05) (Figure 5C1,C2,C3). The expression of Rab7 in Group EGFP under hypoxia was higher than that under normoxia, Group rFoxO1 was higher than that in Group EGFP under normoxia and hypoxia exposure (*p* < 0.05) (Figure 5D1). The expression of Atg7 and the ratio of LC3II/I in Group rFoxO1 and K to Q were higher than those in Group EGFP under normoxia, the result in Group EGFP was higher under hypoxia than that under normoxia; the result in Group K to R was lower than that in Groups K to Q and rFoxO1 under hypoxia (*p* < 0.05) (Figure 5D2,E1). The expression of p62 in Group EGFP under hypoxia was lower than that under normoxia, Group rFoxO1 and K to Q was lower than that in Group EGFP under normoxia (*p* < 0.05) (Figure 5E2).

## 4 DISCUSSION

### 4.1 Characteristics of hypoxia‐induced muscle atrophy

The phenomenon of muscle atrophy induced by hypoxia was first discovered in mountaineering.^32^ The decline of food intake in a hypoxic environment was initially thought to be an important reason for the induction of muscle atrophy,^17^, ^33^ but later studies found that providing subjects with favourite foods and encouraging eating did not prevent skeletal muscle loss.^34^ Our study also found that the reduction of food intake in rats caused by hypoxia occurred in the early stage, and the food intake of each group tended to be similar in the later stage, which was inadequate for muscle atrophy (Figure S1). However, the body weight, lean body mass, muscle mass and FCSA of the rats were significantly reduced after 4 weeks of hypoxic exposure.

The loss of skeletal muscle mass is closely related to oxygen concentration. The partial pressure of oxygen (PO_2_) in air changes the blood oxygen saturation (SaO_2_) and the oxygen dissociation curve, by affecting the arterial PO_2_. Some studies believed that SaO_2_ could be used instead of oxygen concentration (altitude) to define the ‘muscle atrophy zone’. At the altitude of 4000 m, the slope of the oxygen dissociation curve increased sharply, and this altitude was considered to contribute to muscle atrophy.^35^ Therefore, we chose to simulate the oxygen concentration at an altitude of 4000 m in vivo. Since the cells and the body do not correspond to the perception of hypoxia, the oxygen concentration in in vivo research intervention cannot be directly related to in vitro,^36^ and the relationship between the two should be further explored. Studies have found that 2% O_2_ induces a more significant reduction in the diameter of C2C12 myotubes than 5% and 20% O_2._ ^37^ We also compared the effects of 1%, 3% and 5% O_2_ and interventions for 6, 12 and 24 h on the diameter of L6 myotubes (Figure S2), and finally decided to intervene with 1% O_2_ for 6 h as the hypoxic conditions in vitro.

Furthermore, each type of muscle fibres has its sensitivity to hypoxia. In our study, the degree of atrophy of fast‐twitch (EDL) and mixed‐twitch (GAS) muscles were higher than that of SOL caused by hypoxia. Hypoxia inducible factor‐1α (HIF‐1α, key factor stimulated by hypoxia) may be highly expressed in fast‐twitch fibres, which lead to the increase in the expression of the glucocorticoid receptor (GR) and its target genes^38^; meanwhile, glucocorticoid (GC) inhibits the expression of vascular endothelial growth factor (VEGF),^39^ causing decreased angiogenesis, leading to poor adaptation of fast‐twitch fibres to hypoxia.^40^

### 4.2 Effects of resistance training under hypoxia on FoxO1 and its acetylation

Among the various forms of exercise, resistance training stimulates the skeletal muscles the most. Different forms and intensities of resistance training promote skeletal muscle hypertrophy in normoxia.^41^ The effect of resistance training in relieving aging and diabetes‐induced muscle atrophy has been confirmed, but its effect in relieving hypoxia‐induced atrophy needs further study. Studies have found that a variety of exercise modalities, including resistance training, improve muscle atrophy in patients with COPD. Weight‐bearing ladder training promotes hypertrophy under the condition of increasing load, which is the closest training method to human resistance exercise known so far.^42^ Our study also confirmed that resistance training increases the lean body mass of rats in hypoxia. Resistance training‐induced muscle hypertrophy may also be fibre‐selective, with fast‐twitch fibres more likely to hypertrophy than slow‐twitch in normoxia.^43^, ^44^ Wet mass and FCSA of fast‐twitch muscles (EDL) were significantly increased after resistance training under hypoxia in our study, so we chose EDL as the object in the follow‐up research.

As a nuclear transcription factor, nuclear localisation was previously thought to be a prerequisite for FoxO1 to play a functional role. But recent studies have found that cytoplasmic FoxO1 may lead to different cell survival outcomes in a different way other than nuclear transcription, and FoxO1 acetylation/deacetylation modification may play a pivotal role in this process.^23^ The acetylation condition of FoxOs is generally regulated by deacetylase. SIRT2 is an inducible NAD^+^‐dependent deacetylase of Class III histones, mainly present in the cytoplasm. SIRT2 interacts directly with FoxO1 In the cytoplasm, but dissociates from FoxO1 under external stimuli, resulting in FoxO1 acetylation.^45^

NAD^+^ is an essential co‐substrate of sirtuin, and the balance of oxidized form (NAD^+^) and reduced form (NADH) determines the function of NAD^+^‐dependent enzymes. Nicotinamide phosphoribosyl transferase (NAMPT), the rate‐limiting enzyme in the NAD^+^ pathway of skeletal muscle, provides a possibility to study exercise regulation of SIRT2.^46^, ^47^, ^48^ Our study demonstrated that FoxO1 and Ac‐FoxO1 protein expression was increasing after hypoxia exposure, as well as FoxO1 nuclear localisation, while SIRT2 activity was reducing and Ac‐FoxO1 expression was promoting in the cytoplasm. Resistance training under hypoxia reduces the protein expression and nuclear localisation of FoxO1, increases SIRT2 activity, and reduces the expression of Ac‐FoxO1 in the cytoplasm.

### 4.3 FoxO1 and its acetylation mediate ALP in skeletal muscle

The increasing rate of muscle protein breakdown is a key point for hypoxia inducing atrophy. Resistance training mainly enhances muscle protein synthesis to promote muscle hypertrophy in normoxia or in healthy bodies; in hypoxia, aging and various disease‐induced atrophy organisms, inhibition of skeletal muscle protein breakdown may be the key to its anti‐atrophic effect.^49^

In our study, UPS may not play a key regulatory role. MuRF1 and Atrogin‐1 specifically label muscle proteins for targeted degradation by the 26S proteasome, which were once considered to be key proteins in the activation of UPS. Then after more E3 ubiquitin protein ligases were discovered, MuRF1 and Atrogin‐1 were more considered to be the marker of muscle atrophy due to their specificity in skeletal muscle. The ubiquitin of proteins could be determined by measuring the ubiquitination of the whole protein. Our study found that hypoxia increased the expression of Atrogin‐1 and MuRF1 in EDL, but did not change the level of ubiquitination, and resistance training under hypoxia did not change UPS levels. The overexpression of the active form of FoxO1 in C2C12 myocytes did not alter basal levels of MuRF1 or Atrogin‐1, and overexpression of Atrogin‐1 in skeletal muscle is not sufficient to induce atrophy.^15^, ^16^ Therefore, ALP may play a more important regulatory role in muscle proteolysis. When ALP is inhibited, the polyubiquitinated protein degradation is disrupted, and the accumulation of proteins to be degraded continues to increase, and UPS will be compensatory activated. Moreover, ALP has stronger muscle atrophy effect than UPS.^50^

ALP is an important pathway for protein breakdown, and its processes include the initiation of autophagy, the formation of autophagosomes, and the formation and degradation of autophagolysosomes.^51^ The formation of autophagosomes requires the participation of LC3. The precursor of LC3 can be cleaved to form a soluble mature LC3I, which is then lipidated by Atg7 and combined with phosphatidyl ethanolamine (PE) to form a lipid‐soluble complex LC3II‐PE, marking the formation of autophagosomes.^52^ Autophagic flux could be measured by LC3II/I.^53^ p62 is an adapter protein that forms protein aggregates and is specifically bound to the autophagosome membrane. It is a selective and specific substrate for autophagy, binding to LC3 and recruits ubiquitinated proteins to be transported to the autophagosome, where it is transported to the autophagosome, and degraded in autophagic lysosomes, and decreased p62 expression indicates increased autophagy.^54^ Rab7 plays an important regulatory role in autophagy mediated by FoxO1, mainly mediating autophagosome and lysosome fusion in late autophagy stage.^55^, ^56^ Loss of LC3 and Atg7 could directly induce muscle atrophy, especially complex formation with Atg7 is required for the acetylation of FoxO1.^23^, ^57^

Our results showed that hypoxia could increase ALP in rats' EDL; simulated FoxO1 acetylation significantly could reduce myotube diameter in both normoxia and hypoxia, and simulated deacetylation might increase myotube diameter in hypoxia. Hypoxia increases the expression of ALP‐related proteins in myotubes. FoxO1 overexpression increases the expression of Rab7, whereas the altering acetylation status has no effect on Rab7. FoxO1 overexpression and simulated FoxO1 acetylation both increased the expression of Atg7 in normoxia, whereas simulated FoxO1 deacetylation decreased the expression of Atg7 in hypoxia. The LC3II/I ratio was used to reflect the overall level of autophagy, and the results were similar to the expression of Atg7, suggesting that Atg7 plays a crucial role in the regulation of autophagy in skeletal muscle by FoxO1 acetylation. The FoxO1 acetylation in vitro is confirmed in our study. Considering factors such as the difference of maintenance time of in vivo injected plasmids and training time, we did not conduct in in vivo, which needs to be improved in follow‐up studies.

Cytoplasmic FoxO1‐mediated Atg7‐promoted autophagy has been reported in cancer cells.^23^ After serum starvation or hydrogen peroxide (H_2_O_2_) stimulation of HCT116 cells, the interaction of FoxO1 with SIRT2 was significantly attenuated, and the acetylation level of cytoplasmic FoxO1 was increased, but the phosphorylation and ubiquitination of FoxO1 were not observed.^58^ The interaction between FoxO1 and ATGs was detected, which could only be found between Atg7 and FoxO1 enhanced by serum starvation. Endogenous Atg7 interacts with FoxO1 in SIRT2 mutants (losing sirtuin activity), suggesting that FoxO1 acetylation is a modification required for Atg7 binding.^23^

## 5 CONCLUSIONS

Hypoxia could promote the development of muscle atrophy by activating FoxO1‐mediated autophagy, in which nuclear FoxO1‐mediated Rab7 and cytoplasmic Ac‐FoxO1‐mediated Atg7 are involved in regulating this process; however, resistance training could inhibit autophagy‐induced atrophy by inhibiting the nuclear transcriptional activity of FoxO1 and the dissociation of cytoplasmic FoxO1 and SIRT2 to reduce deacetylation.

## AUTHOR CONTRIBUTIONS

**Pengyu Fu:** Writing – original draft (lead). **Rongxin Zhu:** Methodology (lead). **Weiyang Gao:** Writing – review and editing (supporting). **Lijing Gong:** Resources (lead).

## FUNDING INFORMATION

This work was supported financially by grants from the China Fundamental Research Funds for the Central Universities (Beijing Sport University File No. 2019PT003 and Northwestern Polytechnical University File No. 23GH030634).

## CONFLICT OF INTEREST STATEMENT

All authors disclosed no relevant relationships.

## Supporting information


Data S1:

## Data Availability

The datasets used and/or analysed during the current study are available from the corresponding author on reasonable request.
