# Supplementary material for: Effects of resistance training on alleviating hypoxia‐induced muscle atrophy: Focus on acetylation of FoxO1
Source: J Cell Mol Med. 2023 Dec 27;28(3):e18096. doi: 10.1111/jcmm.18096 (PMC10844693; doi:10.1111/jcmm.18096)
Supplement: Supplementary file 1 — Data S1: [file JCMM-28-e18096-s001.docx]

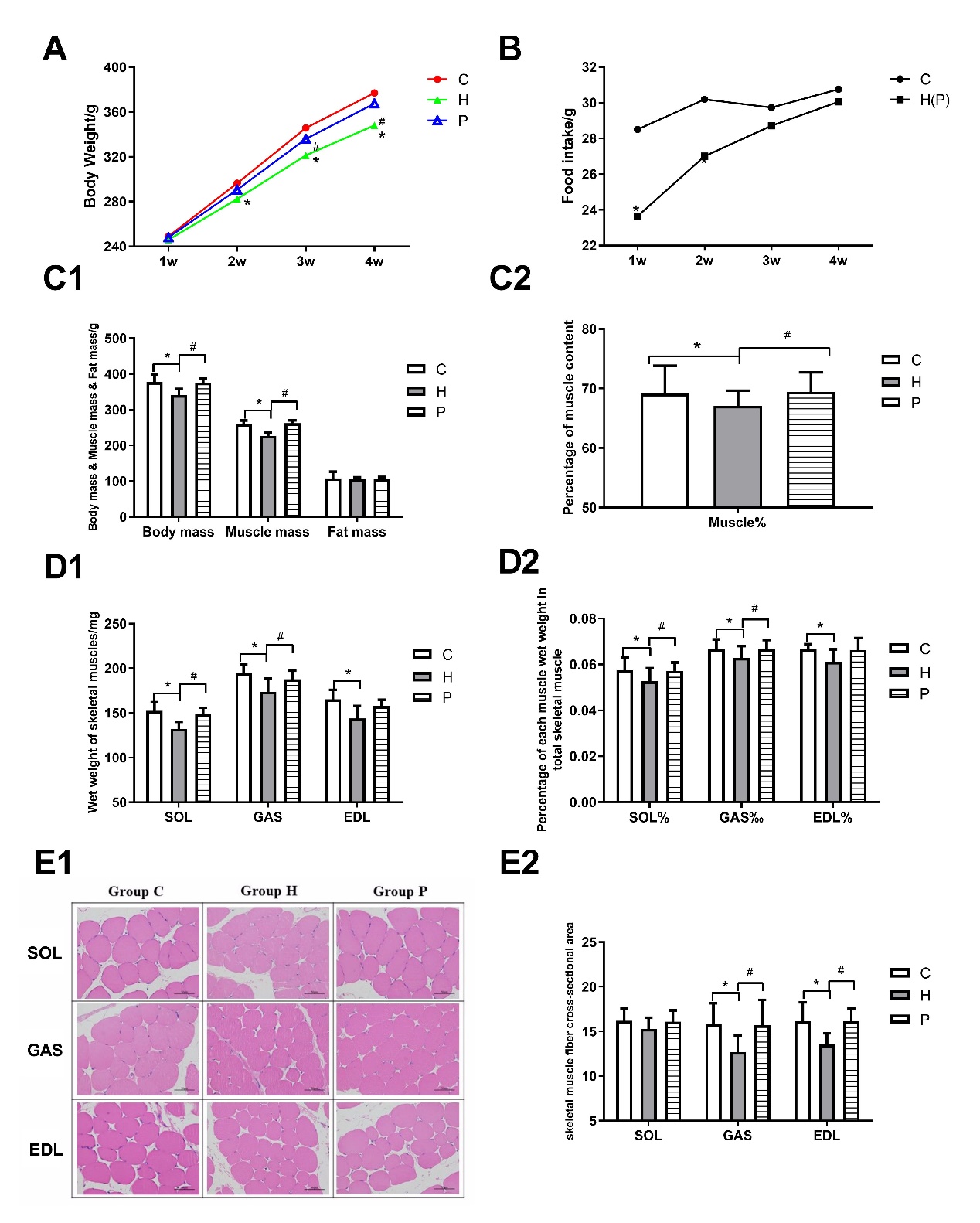


**SUPPLEMENTARY FIGURE 1 |** Effects of changes in food intake on body composition and muscle atrophy in rats under hypoxia. The weekly body weight (A) and food intake during the intervention (B) (*n*=6). The body mass, lean body mass and the fat mass (C1), the percentage of lean body mass (C2), the percentage of SOL, GAS and EDL wet weight (wet weight / lean body mass) (D) after the intervention (*n*=6). HE staining (E1) and the FCSA (E2) of SOL, GAS and EDL after the intervention (*n*=3). ^*^ Significant different from Group C; ^#^ Significant different from Group H (*p* < 0.05). d, day; w, week; g, gram; SOL, soleus muscle; GAS, gastrocnemius muscle; EDL, extensor digitorum longus muscle; C, normoxic control; H, hypoxic control; P, normoxia paired with hypoxic diet group (The food intake of group P is the food intake of group C the day before).


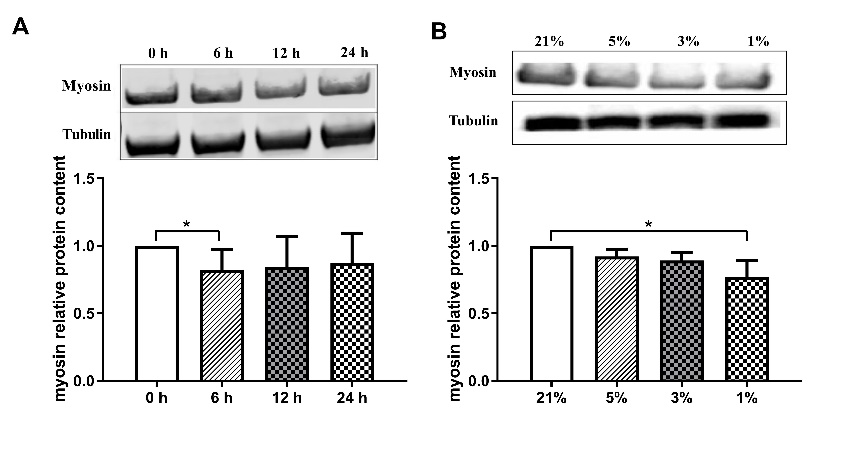


**SUPPLEMENTARY FIGURE 2** | The expression protein of Myosin in myotubes under different hypoxia time and oxygen concentration (*n*=3). The results showed that exposure to 1% oxygen for 6 hours was the optimal condition for inducing myotube atrophy.
